# Supplementary material for: Microstructural changes precede depression in patients with relapsing-remitting Multiple Sclerosis
Source: Commun Med (Lond). 2023 Jun 22;3:90. doi: 10.1038/s43856-023-00319-4 (PMC10287644; doi:10.1038/s43856-023-00319-4)
Supplement: Supplementary file 1 — Description of Additional Supplementary Files [file 43856_2023_319_MOESM1_ESM.pdf]

Description of Additional Supplementary Files

File Name: Supplementary Data

Description: Code used in analysis
