# Supplementary material for: Microstructural changes precede depression in patients with relapsing-remitting Multiple Sclerosis
Source: Commun Med (Lond). 2023 Jun 22;3:90. doi: 10.1038/s43856-023-00319-4 (PMC10287644; doi:10.1038/s43856-023-00319-4)
Supplement: Supplementary file 3 — Supplementary information [file 43856_2023_319_MOESM3_ESM.pdf]

Supplementary information

Supplementary Table 1 Average FWF index of segmented subcortical nuclei and whole white and grey matter in arbitrary units before and after erosion

Structure	Baseline (a.u.) before erosion	1-year follow-up (a.u.) before erosion	Baseline (a.u.) after erosion	1-year follow-up (a.u.) after erosion
**Thalamus**	0.24 ± 0.04	0.24 ± 0.05	0.26 ± 0.04	0.26 ± 0.05
**Caudate**	0.25 ± 0.05	0.25 ± 0.06	0.23 ± 0.05	0.24 ± 0.06
**Putamen**	0.13 ± 0.07	0.13 ± 0.09	0.22 ± 0.08	0.23 ± 0.08
**Pallidum**	0.13 ± 0.07	0.13 ± 0.09	0.25 ±0.08	0.26 ±0.08
**Hippocampus**	0.24 ± 0.03	0.24 ± 0.05	0.26 ± 0.04	0.26 ± 0.05
**Amygdala**	0.26 ± 0.06	0.26 ± 0.09	0.23 ± 0.08	0.23 ± 0.08
**Accumbens**	0.15 ± 0.05	0.16 ± 0.08	0.18 ± 0.08	0.19 ± 0.07
**Whole white matter**	0.25 ± 0.01	0.25 ± 0.01	N.A.	N.A.
**Whole grey matter**	0.30 ± 0.02	0.30 ± 0.02	N.A.	N.A.

No diffusion data was available for the 3-year follow-up.

Supplementary Table 2 Average volumes of segmented subcortical nuclei in mm^3 for all participants at the individual timepoints.

Structure	Baseline (mm^3)	1-year follow-up (mm^3)	2-year follow-up (mm^3)
**Thalamus**	8493 ± 967.0	8449 ± 874.6	8331 ± 858.7
**Caudate**	3460 ± 459.6	3443 ± 430.4	3402 ± 404.2
**Putamen**	5044 ± 609.5	5021 ± 538.9	5012 ± 474.7
**Pallidum**	1865 ± 217.2	1845 ± 224.8	1845 ± 179.1
**Hippocampus**	3470 ± 523.5	3420 ± 496.4	3358 ± 455.7
**Amygdala**	1329 ± 207.7	1338 ± 208.7	1273 ± 220.3
**Accumbens**	496 ± 107.6	493 ± 98.4	484 ± 102.1

Supplementary Table 3: Volume changes of the deep gray matter nuclei between TP1 and TP2 in percent of total volume.

Thalamus	Caudate	Putamen	Pallidum	Hippocampus	Amygdala	Accumbens
0 ± 0.5%	0 ± 0.3%	0 ± 0.5%	-1 ± 0.75%	-1 ± 0.9%	+2 ± 1.3%	0 ± 1.0%

Supplementary Table 4 Volume changes of the deep gray matter nuclei between TP1 and TP3 in percent of total volume

Thalamus	Caudate	Putamen	Pallidum	Hippocampus	Amygdala	Accumbens
-2 ± 0.35%	-1 ± 0.2%	-1 ± 0.4%	0 ± 0.3%	-2 ± 1.9%	-4 ± 1.2%	-3 ± 0.9%
