# Supplementary material for: Microstructural changes precede depression in patients with relapsing-remitting Multiple Sclerosis
Source: Commun Med (Lond). 2023 Jun 22;3:90. doi: 10.1038/s43856-023-00319-4 (PMC10287644; doi:10.1038/s43856-023-00319-4)
Supplement: Supplementary file 4 — Reporting Summary [file 43856_2023_319_MOESM4_ESM.pdf]

nature portfolio

Corresponding author(s): Frank Riemer

Last updated by author(s): May 12, 2023

Reporting Summary

Nature Portfolio wishes to improve the reproducibility of the work that we publish. This form provides structure for consistency and transparency in reporting. For further information on Nature Portfolio policies, see our Editorial Policies and the Editorial Policy Checklist.

Statistics

For all statistical analyses, confirm that the following items are present in the figure legend, table legend, main text, or Methods section.

n/a	Confirmed	
☐	☒	The exact sample size (*n*) for each experimental group/condition, given as a discrete number and unit of measurement
☐	☒	A statement on whether measurements were taken from distinct samples or whether the same sample was measured repeatedly
☐	☒	The statistical test(s) used AND whether they are one- or two-sided<br>*Only common tests should be described solely by name; describe more complex techniques in the Methods section.*
☐	☒	A description of all covariates tested
☐	☒	A description of any assumptions or corrections, such as tests of normality and adjustment for multiple comparisons
☐	☒	A full description of the statistical parameters including central tendency (e.g. means) or other basic estimates (e.g. regression coefficient) AND variation (e.g. standard deviation) or associated estimates of uncertainty (e.g. confidence intervals)
☐	☒	For null hypothesis testing, the test statistic (e.g. *F*, *t*, *r*) with confidence intervals, effect sizes, degrees of freedom and *P* value noted<br>*Give P values as exact values whenever suitable.*
☒	☐	For Bayesian analysis, information on the choice of priors and Markov chain Monte Carlo settings
☐	☒	For hierarchical and complex designs, identification of the appropriate level for tests and full reporting of outcomes
☐	☒	Estimates of effect sizes (e.g. Cohen's *d*, Pearson's *r*), indicating how they were calculated

Our web collection on statistics for biologists contains articles on many of the points above.

Software and code

Policy information about availability of computer code

Data collection	None.
Data analysis	Matlab 9.5.0 (the MathWorks, Natick, MA).<br>SPM12 r7771 (UCL, London, UK)<br>FSL 6.0.5.2 (FMRIB, Oxford, UK)<br>Custom Code: FWF calculation, available on private Github repository and can be made available on request.

For manuscripts utilizing custom algorithms or software that are central to the research but not yet described in published literature, software must be made available to editors and reviewers. We strongly encourage code deposition in a community repository (e.g. GitHub). See the Nature Portfolio guidelines for submitting code & software for further information.

Data

Policy information about availability of data

All manuscripts must include a data availability statement. This statement should provide the following information, where applicable:

- Accession codes, unique identifiers, or web links for publicly available datasets
- A description of any restrictions on data availability
- For clinical datasets or third party data, please ensure that the statement adheres to our policy

The data that support the findings of this study are available on request from the corresponding author. The data are not publicly available due to containing

information that could compromise the privacy of research participants. Approval for data sharing is subject to approval by the author's local ethics committee and a formal data sharing agreement.

Human research participants

Policy information about studies involving human research participants and Sex and Gender in Research.

Reporting on sex and gender	Only the biological sex at birth was recorded and information on gender was not collected. In the revision of the manuscript we have made sure to use the term "sex" when referring to the sex at birth.
Population characteristics	Only age was considered as a covariate and was used in the analysis. Age median and age range are given for this longitudinal study.
Recruitment	A total of 65 participants with relapsing-remitting MS as defined by the 2017 revision of the McDonald criteria were recruited at the Department of Neurology, Haukeland University Hospital in this longitudinal study. A total of 46 participants completed the study. A bias may exist in clinical, educational and disease demographics in the group that completed the study as opposed to the ones who did not attend all 3 visits. I can only speculate, but I assume that more severely depressed individuals are less likely to complete the study and that our results could have been stronger if these individuals had indeed attended all visits.
Ethics oversight	Regional Ethics Committee of Western Norway (registration number 2016/31/REK Vest).

Note that full information on the approval of the study protocol must also be provided in the manuscript.

Field-specific reporting

Please select the one below that is the best fit for your research. If you are not sure, read the appropriate sections before making your selection.

☒ Life sciences ☐ Behavioural & social sciences ☐ Ecological, evolutionary & environmental sciences

For a reference copy of the document with all sections, see nature.com/documents/nr-reporting-summary-flat.pdf

Life sciences study design

All studies must disclose on these points even when the disclosure is negative.

Sample size	Sample size was determined by recruitment from clinic over an 11 month period. Out of the 65 recruited subjects, two opted out and withdrew their consent, two did not attend the baseline visit, four did not return after the baseline visit and seven did not attend for the two-year follow up. One subject missed the one-year follow-up but had completed the baseline and two-year follow-up visits, and was not removed from the dataset. A further three subjects had incomplete cognitive scores at the two-year follow-up visit and were therefore excluded. In total, 46 participants had complete data collection at baseline and two-year follow-up and were therefore included in this study.
Data exclusions	No data was excluded.
Replication	All parameters for replication of the results are given and the study can be performed alike or the results re-analysed with the same conclusion.
Randomization	Randomization was not relevant as we do not compare different groups of subjects.
Blinding	Not applicable.

Reporting for specific materials, systems and methods

We require information from authors about some types of materials, experimental systems and methods used in many studies. Here, indicate whether each material, system or method listed is relevant to your study. If you are not sure if a list item applies to your research, read the appropriate section before selecting a response.

Materials & experimental systems

n/a	Involved in the study
☒	☐ Antibodies
☒	☐ Eukaryotic cell lines
☒	☐ Palaeontology and archaeology
☒	☐ Animals and other organisms
☐	☒ Clinical data
☒	☐ Dual use research of concern

Methods

n/a	Involved in the study
☒	☐ ChIP-seq
☒	☐ Flow cytometry
☐	☒ MRI-based neuroimaging

Clinical data

Policy information about clinical studies

All manuscripts should comply with the ICMJE guidelines for publication of clinical research and a completed CONSORT checklist must be included with all submissions.

Clinical trial registration	NA
Study protocol	NA
Data collection	NA
Outcomes	NA

Magnetic resonance imaging

Experimental design

Design type	NA
Design specifications	NA
Behavioral performance measures	NA

Acquisition

Imaging type(s): Structural and diffusion

Field strength: 3T

Sequence & imaging parameters:

The parameters were as follows:

3D volumetric T1w sagittal volume, TE/TR/TI = 2.28 ms/1.8 s/900 ms, acquisition matrix = 256 x 256 x 192, FOV = 256 x 256 mm2, slice thickness = 1 mm, 200 Hz/px readout bandwidth and total acquisition duration of 7.4 minutes.

2D axial T2w volume, TE/TR = 100.0 ms/6.0 s, acquisition matrix = 512 x 384, FOV = 220 x 220 mm2, slice thickness = 4 mm, 220 Hz/px readout bandwidth and total acquisition duration of 2.1 minutes.

3D volumetric T2-FLAIR sagittal volume, TE/TR/TI = 386 ms/5.0 s/1600 ms, acquisition matrix = 256 x 256 x 192, FOV = 256 x 256 mm2, slice thickness = 1 mm, 751 Hz/px readout bandwidth and total acquisition duration of 6.2 minutes.

Area of acquisition: Whole brain.

Diffusion MRI ☒ Used ☐ Not used

Parameters: 2D axial DWI with 6 diffusion-unweighted volumes and 4 different diffusion-weighted volumes with b-values of 200 (3 directions), 500 (6 directions), 1000 (30 directions) and 2500 (30 directions) s / mm2, TE/TR = 82 ms/9 s, acquisition matrix = 128 x 128, FOV = 256 x 256 mm2, slice thickness = 2 mm, 72 slices, 1500 Hz/px readout bandwidth and total acquisition duration of 11.4 minutes. No respiratory gating was used.

Preprocessing

Preprocessing software: T2 FLAIR images were co-registered to the T1w images within the same imaging session using SPM12 (UCL, UK). Diffusion-weighted images were motion corrected, masked and eddy current corrected using FSL 6.0.1 (the University of Oxford, UK). FWF maps were created in native space using an in-house routine.

Normalization: For the general linear model (GLM) analysis, individual T1w images were transformed into standard space based using non-linear transformations in SPM12.

Normalization template	MNI152 T1w template.
Noise and artifact removal	Diffusion-weighted images were motion corrected, masked and eddy current corrected using FSL 6.0.1 (the University of Oxford, UK).
Volume censoring	No volume censoring as this was not a fMRI study.

Statistical modeling & inference

Model type and settings	NA
Effect(s) tested	NA

Specify type of analysis: ☐ Whole brain ☐ ROI-based ☐ Both

Statistic type for inference (See Eklund et al. 2016)	NA
Correction	NA

Models & analysis

n/a	Involved in the study
☒	☐ Functional and/or effective connectivity
☒	☐ Graph analysis
☐	☒ Multivariate modeling or predictive analysis

Multivariate modeling and predictive analysis	Structural equation modeling (SEM) was set up using a partial least squares algorithm (PLS-SEM). For this, the 18 measurement variables belonging to the FWF mean values of each subcortical region and individual test and questionnaire scores were grouped into the following five latent constructs: FWF in subcortical region (including thalamus, caudate, putamen, pallidum, amygdala and accumbens), EDSS, BICAMS (including SDMT, CVLT-II and BVMT-r), FSMC (including cognitive, motor and total fatigue scores) and HADS (including an anxiety, depression and a total anxiety/depression score). The model was set up as a formative measurement model.
